# How can neuromorphic hardware attain brain-like functional capabilities?

**DOI:** 10.1093/nsr/nwad301

**Published:** 2023-12-01

**Authors:** Wolfgang Maass

**Affiliations:** Computer Science and Biomedical Engineering, Graz University of Technology, Austria

## Abstract

The author provides 4 design principles of how to make cortical microcircuits into neuromorphic hardwares, shedding light for the next generation neuromorphic hardware design.

Research on neuromorphic computing is driven by the vision that we can emulate brain-like computing capability, learning capability, and energy-efficiency in novel hardware. Unfortunately, this vision has so far been pursued in a half-hearted manner. Most current neuromorphic hardware (NMHW) employs brain-like spiking neurons instead of standard artificial neurons. This is a good first step, which does improve the energy-efficiency of some computations (see [1] for one of many examples). But current architectures and training methods for networks of spiking neurons in NMHW are largely copied from artificial neural networks. Hence it is not surprising that they inherit many deficiencies of artificial neural networks, rather than attaining brain-like functional capabilities.

Of course, the brain is very complex, and we cannot implement all its details in NMHW. Instead, we need to focus on principles that are both easy to implement in NMHW and likely to support brain-like functionality. The goal of this article is to highlight some of them.

Although other areas of the brain, such as the hippocampus, provide a rich source of inspiration for NMHW with new functional capabilities, I can discuss here only one brain area, and I have chosen the neocortex, a brain area that is central to its computational prowess. The neocortex can be seen in first approximation as a thin sheet of neurons that is structured like a tapestry, i.e. it is stitched together from repeating local circuit modules which one commonly refers to as cortical microcircuits (CMs) (see [2] and [3]). Porting the functionally most relevant design principles of CMs into NMHW is an attractive and feasible target for the next generation of NMHW design.

**Design Principle 1: CMs consist not just of one or two, but over 100 genetically different types of spiking neurons with different computational roles.** This principle implies that the architecture of CMs is fundamentally different from the randomly connected networks, typically consisting of one or two types of spiking neurons, that are commonly implemented in NMHW. In fact, the sophisticated and genetically encoded structure of CMs is more reminiscent of highly structured digital circuits such as CPUs. However, they employ small populations of units, rather than single units, for specific computational roles, and are therefore more robust to failures of individual units. In fact, CMs have in some sense an even more sophisticated structure than CPUs because they employ a substantially larger repertoire of different units. One prominent example is genetically distinct types of excitatory neurons (pyramidal cells) that report specific prediction errors for top-down predictions of visual flow [4].

Another instructive example is different types of inhibitory neurons, which in CMs play a role for computation and learning that is very different from that in current NMHW. There one commonly views inhibitory neurons as clones of excitatory neurons whose outputs have a negative sign. This is convenient for emulating arithmetical computations with negative and positive values, or artificial neural networks. But in the brain, inhibitory neurons play quite different roles. Think for example of populations of specific types of excitatory neurons as being ‘experts’ for specific knowledge or tasks, and inhibitory neurons as controllers which determine which ‘expert’ is allowed to impact the computational task at hand, and which ‘expert’ is allowed to improve its competence by learning from the current task through synaptic plasticity. According to [3,5,6], one type of inhibitory neurons (PV cells) can veto the firing of selected excitatory neurons, which they target through dozens of synapses on their soma. Another inhibitory neuron type (SOM cells) blocks activity and synaptic plasticity in selected input regions (dendrites) of specific excitatory neurons. A third inhibitory neuron type (VIP cells) inhibits both of the previously mentioned types of inhibitory neurons. In other words, VIP cells are disinhibitory: They can remove the inhibitory lock for firing and/or synaptic plasticity for specific populations of excitatory neurons. Their function points to a fundamental difference between CMs on one hand, and current artificial neural networks and NMHW on the other hand: Computation and learning are commonly treated as 2 distinct processes in NMHW, but are intertwined in sophisticated ways in CMs. Most *in-vivo* results on synaptic plasticity point to the involvement of one or several gating factors [7] that result from the firing of specific populations of neurons. Some of these gating factors, such as disinhibition through VIP cells, is automatically local. However, neuromodulatory gating signals were recently found to be much more target specific than had been previously assumed. Such a target- and context-specific local regulation of synaptic plasticity is likely to alleviate problems with continual learning that exist in current NMHW, but not in brains.

The genetic code specifies connection probabilities between each pair of the over 100 neuron types [5,6,8,9]. Our analysis [10] shows that this enables the genetic code to program specific computational capabilities into CMs that do not require any learning, as postulated by [11]. In fact, in [10] we had not even exploited the fact that different neuron types can have very different firing properties, which is likely to enhance the innate computing capabilities of CM. Another next step will be to study how desirable learning biases can be programmed in this way into NMHW, thereby enabling them to learn from only a few examples.

**Design Principle 2: CMs employ soft rank order coding, i.e., a form of temporal coding, using very sparse activity.** The idea to use spike timing for encoding analog values had already been proposed a long time ago. However, rank order coding with single spikes is not robust to the deletion or addition of single spikes. The neocortex employs a less brittle rank order coding scheme: instead of single spikes, the rank order of the times of peak firing activity of different neurons is used to encode information. This type of soft rank order coding, which can be reproduced in CM-like neural network models [9], is robust to timing jitter, and to the deletion or addition of single spikes. Rank order coding is of particular interest for NMHW because it has recently been shown to provide close-to-optimal energy efficiency for neural coding (see the Supplement).

**Design Principle 3: CMs employ a sophisticated combination of segregation and integration of information over neurons.** Segregation of information in specialized populations of neurons and the integration of their contribution to a coherent network output has been argued to be characteristic for the organization of brain computations [12]. The analysis of [9] shows that this principle can be reproduced in CM models. Specifically, projection neurons that report network outputs of CMs turn out to be highly sensitive to the firing activity of small sets of ‘expert neurons’. Furthermore, this holds in spite of the remarkable level of noise to which CM computations are subjected. On the other hand, we found that those spiking and non-spiking neural network architectures that have so far been implemented in NMHW do not have this property [9]. Note that functional segregation tends to enhance continual learning, since not every neuron gets involved in every computational process.

**Design Principle 4: computing capabilities of CMs are shaped by diverse local synaptic plasticity rules.** One commonly implements learning in NMHW with a single rule for synaptic plasticity, such as spike-timing dependent plasticity (STDP), or with a single global learning scheme such as back propagation through time (BPTT). But the power of STDP for installing computing capabilities in NMHW is quite limited, and BPTT is not suitable for on-chip learning. Hence new learning methods need to be explored for NMHW. The brain suggests to use different plasticity rules for different types of neurons and different forms of learning. Furthermore, most of the biologically found rules do not require a teacher. Many of them do not even depend on postsynaptic firing, and are therefore fundamentally different from STDP and other Hebb-like plasticity rules (see [7] and further references in the Supplement). The rich repertoire of synaptic plasticity rules in CMs is likely to provide a functionally powerful alternative to BPTT that is suitable for on-chip learning in NMHW. This diverse set of local plasticity rules is also likely to enhance the emergence of neurons that become selective for complex combinations of features in sensory input streams, thereby providing a substantially more sparsely active but still functionally powerful alternative to convolutional neural networks for visual processing.

Implementing selected facets of these four design principles in NMHW provides a road map for reproducing in NMHW more energy efficiency through sparser firing, as well as more powerful brain-like computing and learning capabilities. These design principles are especially suited for creating autonomously learning devices that, like brains, are able to detect and make sense of salient patterns in very high-dimensional multi-modal input streams, and no longer require training with engineered homogeneous data sets in order to become smart.

## Supplementary Material

nwad301_Supplemental_File
