# Supplementary material for: How can neuromorphic hardware attain brain-like functional capabilities?
Source: Natl Sci Rev. 2023 Dec 1;11(5):nwad301. doi: 10.1093/nsr/nwad301 (PMC10989294; doi:10.1093/nsr/nwad301)
Supplement: nwad301_Supplemental_File [file nwad301_supplemental_file.pdf]

Supplementary Information for the Perspective Article "How can neuromorphic hardware attain brain-like functional capabilities?"

Wolfgang Maass

16th December 2023

We point here to literature that provides a deeper understanding of the four design principles that are sketched in the article.

To Design Principle 1: [1] is an important source for information about the structure of the neocortex and cortical microcircuits. It is still very readable and relevant. Further insight into functional specialization of genetically different types of pyramidal cells are provided by [2, 3]. Experimental evidence for the fact that excitatory neurons (pyramidal cells) are generically under an inhibitory lock, and require disinhibition for firing was provided in [4].

Experimental data on gating of synaptic plasticity in CMs are reviewed in [5, 6]. Data on the role of disinhibition for synaptic plasticity can be found for example in [7]. Experimental data on the diversity of dopamine signals were provided by [8]. Note that the biologically found gating signals can also be viewed as learning signals for e-prop [9], and therefore support also some forms of network gradient descent learning that can be implemented in NMHW such as Loihi.

To Design Principle 2: The vision to compute with rank-order coding of single spikes had apparently been first proposed by [10]. In [11] it was rigorously proven that this type of rank-order coding enables spiking neural networks to emulate ANNs with single spikes, rather than firing rates. Apparently the first experimental evidence for the more noise robust biologically found type of rank order coding was provided in [12]. Data from many more brain areas are provided in [13]. The energy efficiency of rank order coding was recently analyzed in [14]. Properties of CMs that support soft rank order doing and a quantitative measure for rank order coding can be found in [15].

To Design Principle 3: Functional segregation on the level of single neurons of the neocortex was discussed for example in [16, 17]. A quantitative measure for functional segregation was introduced in [15], and it was shown that CM-like NN models, but not randomly connected neural networks exhibit segregation and integration of information.

To Design Principle 4: The diversity of synaptic plasticity rules found in the neocortex is reviewed for example in [18, 5, 6, 19]. Specific examples of neural coding properties of neurons that are not likely to emerge through BPTT are discussed for example in [20, 21, 22, 23].

References

[1] Vernon B Mountcastle. Perceptual neuroscience: The cerebral cortex. *Harvard University Press*, 1998.

[2] Sean M O'Toole, Hassana K Oyibo and Georg B Keller. Molecularly targetable cell types in mouse visual cortex have distinguishable prediction error responses. *Neuron* 2023; **111**: 2918–2928.

[3] Simon Musall *et al.* Pyramidal cell types drive functionally distinct cortical activity patterns during decision-making. *Nature neuroscience* 2023; **26**: 495–505.

[4] Bilal Haider, Michael Häusser and Matteo Carandini. Inhibition dominates sensory responses in the awake cortex. *Nature* 2013; **493**: 97–100.

[5] Jeffrey C Magee and Christine Grienberger. Synaptic plasticity forms and functions. *Annual review of neuroscience* 2020; **43**: 95–117.

[6] Ronan Chéreau *et al.* Circuit mechanisms for cortical plasticity and learning. *Seminars in cell & developmental biology* 2022; **125**: 68–75.

[7] Johannes J Letzkus, Steffen BE Wolff and Andreas Lüthi. Disinhibition, a circuit mechanism for associative learning and memory. *Neuron* 2015; **88**: 264–276.

[8] Ben Engelhard *et al.* Specialized coding of sensory, motor and cognitive variables in VTA dopamine neurons. *Nature* 2019; **570**: 509–513.

[9] Guillaume Bellec *et al.* A solution to the learning dilemma for recurrent networks of spiking neurons. *Nature communications* 2020; **11**: 3625.

[10] Simon J Thorpe. Spike arrival times: A highly efficient coding scheme for neural networks. *Parallel processing in neural systems* 1990; 91–94.

[11] Wolfgang Maass. On the computational complexity of networks of spiking neurons. *Advances in neural information processing systems* 1994; **7**.

[12] Laura N Driscoll *et al.* Dynamic reorganization of neuronal activity patterns in parietal cortex. *Cell* 2017; **170**: 986–999.

[13] Sue Ann Koay *et al.* Sequential and efficient neural-population coding of complex task information. *Neuron* 2022; **110**: 328–349.

[14] Kwabena Boahen. Dendrocentric learning for synthetic intelligence. *Nature* 2022; **612**: 43–50.

[15] Guozhang Chen, Franz Scherr and Wolfgang Maass. Data-based large-scale models provide a window into the organization of cortical computations. *bioRxiv* 2023; 2023–04.

[16] Arthur R Houweling and Michael Brecht. Behavioural report of single neuron stimulation in somatosensory cortex. *Nature* 2008; **451**: 65–68.

[17] Henry WP Dalgleish *et al.* How many neurons are sufficient for perception of cortical activity? *Elife* 2020; **9**: e58889.

[18] Rylan S Larsen and P Jesper Sjöström. Synapse-type-specific plasticity in local circuits. *Current opinion in neurobiology* 2015; **35**: 127–135.

[19] Amanda R McFarlan *et al.* The plasticitome of cortical interneurons. *Nature Reviews Neuroscience* 2023; **24**: 80–97.

[20] William E Vinje and Jack L Gallant. Sparse coding and decorrelation in primary visual cortex during natural vision. *Science* 2000; **287**: 1273–1276.

[21] Bruno A Olshausen and David J Field. Sparse coding of sensory inputs. *Current opinion in neurobiology* 2004; **14**: 481–487.

[22] Andreas J Keller, Morgane M Roth and Massimo Scanziani. Feedback generates a second receptive field in neurons of the visual cortex. *Nature* 2020; **582**: 545–549.

[23] Mehmet Fişek *et al.* Cortico-cortical feedback engages active dendrites in visual cortex. *Nature* 2023; 1–8.
